# Risk of progression in intermediate age-related macular degeneration among patients using systemic beta-blockers

**DOI:** 10.3389/fopht.2025.1535791

**Published:** 2025-04-14

**Authors:** Brady Hogan, Nihaal Mehta, Anne Strong Caldwell, A. Itzam Marin, Zafar S. Gill, Andres Liske-Cervantes, Marc T. Mathias, Niranjan Manoharan, Alan G. Palestine, Talisa E. de Carlo Forest, Naresh Mandava, Anne M. Lynch, Jennifer L. Patnaik

**Affiliations:** ^1^ University of Colorado School of Medicine, Aurora, CO, United States; ^2^ Department of Ophthalmology, University of Colorado School of Medicine, Anschutz Medical Campus, Aurora, CO, United States

**Keywords:** retina, macula, degeneration, AMD, beto-blockers

## Abstract

**Purpose:**

This study aims to determine whether systemic beta-blocker use over time influences the progression from intermediate to advanced age-related macular degeneration (AMD).

**Methods:**

This prospective cohort study utilized data from the University of Colorado Age-Related Macular Degeneration Registry at the UCHealth Sue Anschutz-Rodgers Eye Center. Patients with intermediate AMD (iAMD) enrolled between October 2014 and November 2021. At enrollment, patient demographics and medication history were recorded. Beta-blocker use was assessed at enrollment and at each follow-up visit. Participants were asked to return annually for imaging, and images were classified as either intermediate AMD or conversion to advanced non-neovascular (NNV) AMD or neovascular (NV) AMD by two vitreoretinal specialists using multimodal imaging. Time to conversion was analyzed using Kaplan–Meier curves for each advanced AMD phenotype and for overall conversion, stratified by beta-blocker status. Progression from intermediate to advanced AMD (NNV or NV) was determined using multimodal imaging, including optical coherence tomography, color fundus photography, and fundus autofluorescence of the posterior pole.

**Results:**

A total of 292 patients were included in the study, with 22.6% using a systemic beta-blocker and 36.6% (*n* = 107) progressing from iAMD to advanced AMD in at least one eye. Patients on a beta-blocker at enrollment were more likely to convert to NV AMD (HR: 1.92 [95% CI: 1.04, 3.55], *p*-value = 0.036), but this association was no longer significant after adjusting for age and treated hypertension. No significant differences were observed in conversion to advanced NNV or any advanced AMD between groups (all *p* > 0.05).

**Conclusions:**

In adjusted analyses, systemic beta-blocker use was not significantly associated with the risk of progression from iAMD to advanced NV or NNV AMD.

## Introduction

Age-related macular degeneration (AMD) is one of the leading causes of vision loss worldwide, affecting 1.98 million Americans (12.6% of those aged 40 years and older) in 2019 (1). The pathophysiology of AMD is complex, with risk factors including cardiovascular disease (particularly hypertension and hyperlipidemia), genetics, diet, age, and smoking history (2). While these associations were debated in studies such as the Beaver Dam Eye Study, they are now widely recognized as contributing to AMD (3). A growing body of research implicates various cellular and molecular pathways, including oxidative damage and innate immunity—particularly the complement pathway—in early AMD development (2). The progression from early to advanced AMD is similarly multifactorial. Advanced NV AMD is characterized by the growth of choroidal neovascular membranes (CNVM), driven primarily by vascular endothelial growth factor (VEGF), a key mediator released by ischemic and hypoxic retinal and choroidal cells in response to AMD-induced damage. VEGF promotes CNVM formation and proliferation, driving the pathological changes characteristic of advanced NV AMD.

Given VEGF’s central role in NV AMD, anti-VEGF therapy was proposed as a treatment for CNVM several decades ago (4). Studies in the early 2000s confirmed its remarkable efficacy, leading to the rapid development of highly effective and safe medications. Anti-VEGF therapy is now the gold standard for NV AMD, revolutionizing the management of this previously blinding condition. Although VEGF pathways are not a primary mechanism in geographic atrophy (GA) development, evidence suggests VEGF may also play a role in advanced NNV AMD. VEGF blockade, for example, may promote GA formation, the hallmark of advanced NNV AMD (5). Thus, understanding VEGF pathway alterations, including those induced by systemic medications, is crucial for understanding both advanced forms of AMD.

Systemic beta-blockers are widely used for nonocular conditions, including hypertension, congestive heart failure, and cardiac arrhythmias. Nonselective beta-blockers (propranolol, sotalol, carvedilol) act on both β1-AR and β2-AR receptors, which are more abundant in the choroid than in the retina. In contrast, selective beta-blockers (atenolol, metoprolol, etc.) primarily target the β1-AR receptor. Beta-blockade has demonstrated anti-VEGF effects (6, 7), which may contribute to its efficacy. For example, topical propranolol is a first-line therapy for certain vascular tumors, such as infantile capillary hemangiomas (8). Previous studies have explored whether beta-blockers could be beneficial in AMD management, potentially by mitigating VEGF-mediated disease pathways or through broader antihypertensive effects (9). However, previous studies have reported conflicting results, showing either no association or a decreased incidence or progression of AMD among patients taking beta-blockers (10–15). These studies have often been limited by cross-sectional design, small sample sizes, or a lack of information on beta-blocker type (nonselective/selective) and duration of use. In this study, we examine the association between systemic beta-blocker use and the progression of intermediate AMD to each form of advanced AMD through a longitudinal analysis, with categorization by beta-blocker type.

## Materials and methods

This prospective study included patients with intermediate AMD (iAMD) recruited into the University of Colorado AMD Registry, previously described in detail (16, 17). Inclusion criteria were as follows: classification as having intermediate AMD at enrollment (October 2014 to November 2021), at least 1 month of ophthalmology follow-up through 15 February 2024, and documented medication use to capture beta-blocker status. Two vitreoretinal specialists graded AMD stage at enrollment using multimodal imaging—optical coherence tomography, color fundus photography, and fundus autofluorescence of the posterior pole—following the Beckman Initiative for Macular Research Committee classification system (18). Baseline patient demographics and medical history, including systemic beta-blocker use, were collected at enrollment. Both neovascular (NV) and non-neovascular (NNV) advanced AMD represent a significant progression from intermediate AMD; therefore, both types of conversion were included in our analysis. Study participants were asked to return annually for blood draws and imaging. Multimodal imaging was used to identify patients who progressed from intermediate to advanced AMD (NV and NNV) based on the Beckman criteria. Conversion type was determined by the first eye to progress from iAMD to either advanced phenotype through 15 February 2024. Conversion to advanced NV AMD was defined by the presence of CNV, while advanced NNV AMD was defined by the presence of GA, based on the Classification of Atrophy Meetings (CAM) consensus.

### Time-varying exposure

Beta-blocker status, medication type, dose, and frequency were recorded during ophthalmology visits from study enrollment until either conversion to advanced AMD (for patients who converted) or the last ophthalmology visit through 15 February 2024 (for those who did not convert). Medication dose and frequency were combined into an average daily amount for each measure.

### Statistical analysis

The primary outcome was a conversion from iAMD to either advanced AMD phenotype—NV or NNV. Basic patient demographics and conversion status were summarized using frequencies and percentages for categorical variables and means and standard deviations for continuous variables. These factors were reported for the entire study population and stratified by beta-blocker use at study enrollment.

The study began at enrollment for all participants and ended either at conversion to advanced AMD or the last appointment with the ophthalmology retina clinic. Kaplan–Meier curves illustrated failure curves for time to conversion to any advanced AMD, as well as separately for NV and advanced NNV. The Kolmogorov-type supremum test assessed the proportional hazards assumption and found no violation. In the first set of models, Cox proportional hazard models estimated the hazard ratio (HR) for conversion based on beta-blocker use at enrollment. Beta-blocker status at enrollment was the primary variable in this analysis. In addition, changes in beta-blocker status and type were summarized using basic frequencies. In the second set of models, Cox proportional hazard modeling with an incorporated beta-blocker is used as a time-varying covariate to evaluate whether changes in exposure influenced the HR. Variables significantly associated with both conversion and beta-blocker use were included as covariates. Multivariable models adjusted for age, with additional adjustment for treated chronic hypertension in the NV model. The Spearman correlation coefficient assessed multicollinearity between beta-blocker use and treated chronic hypertension. To examine potential differences among medications, beta-blockers were categorized into the three most common types (metoprolol succinate, metoprolol tartrate, carvedilol) and all others. These types were compared to the non-use of beta-blockers in the time-varying covariate, age-adjusted model.

## Results

A total of 292 patients with iAMD were included in the study. Baseline characteristics are summarized in **Table 1**. Age differed significantly between study cohorts (*p* = 0.006). Patients were followed for a mean duration of 40.8 months (standard deviation: 25.6). At enrollment, 66 patients (22.6%) were using a systemic beta-blocker. During follow-up, 107 patients (36.6%) progressed to advanced AMD in at least one eye—45 to NV and 62 to advanced NNV. Over half of the study cohort was receiving treatment for chronic hypertension (54.4%) at enrollment. Beta-blocker status and hypertension were moderately correlated (Spearman correlation coefficient: 0.26, *p* < 0.0001).

**Table 1:** Demographic and clinical characteristics of the intermediate AMD patient cohort (*N* = 292).

	Total	Use of beta-blocker at enrollment	Not on beta-blocker at enrollment	p-value
	n (%)	n (%)	n (%)	–
Total (*n*; row %)	–	66 (22.6%)	226 (77.4%)	–
Age (mean; SD)	76.2 (7.0)	78.3 (6.6)	75.6 (7.0)	0.006
Sex				
Male	106 (36.3%)	23 (34.8%)	83 (36.7%)	0.780
Female	186 (63.7%)	43 (65.2%)	143 (63.3%)	
Race/ethnicity				
White	274 (93.8%)	59 (89.4%)	215 (95.1%)	0.183
Black	4 (1.4%)	2 (3.0%)	2 (0.9%)	
Hispanic	8 (2.7%)	3 (4.6%)	5 (2.2%)	
Asian	2 (0.7%)	0	2 (0.9%)	
Other	4 (1.4%)	2 (3.0%)	2 (0.9%)	
History of treated hypertension	159 (54.4%)	52 (78.8%)	107 (47.4%)	< 0.0001
No conversion to advanced AMD	185 (63.4%)	41 (62.1%)	144 (63.7%)	–
Conversion to any advanced AMD	107 (36.6%)	25 (37.9%)	82 (36.3%)	0.813
Conversion to NV	45 (15.4%)	16 (24.2%)	29 (12.8%)	0.062
Conversion to advanced NNV AMD	62 (21.2%)	9 (13.6%)	53 (23.4%)	0.195
Follow-up time (months; mean; SD)	43.2 (26.7)	39.2 (25.6)	44.3 (27.0)	0.139

The types of beta-blockers used among patients are summarized in **Table 2**. Of the 292 patients included in the study, 17 changed their beta-blocker status during follow-up—14 initiated beta-blocker use, while three discontinued it. In addition, 10 patients modified their beta-blocker type or dosage during the study.

**Table 2:** Types of medication among the 82 patients on beta-blockers during the study period.

Selective	n	Nonselective	n
Metoprolol succinate	29	Carvedilol	12
Metoprolol tartrate	22	Propranolol	5
Atenolol	9	Sotalol	2
Nebivolol	6	Labetalol	1

A total of 86 total medications, as some patients were on more than one beta-blocker during the study period.

**Figure 1:**
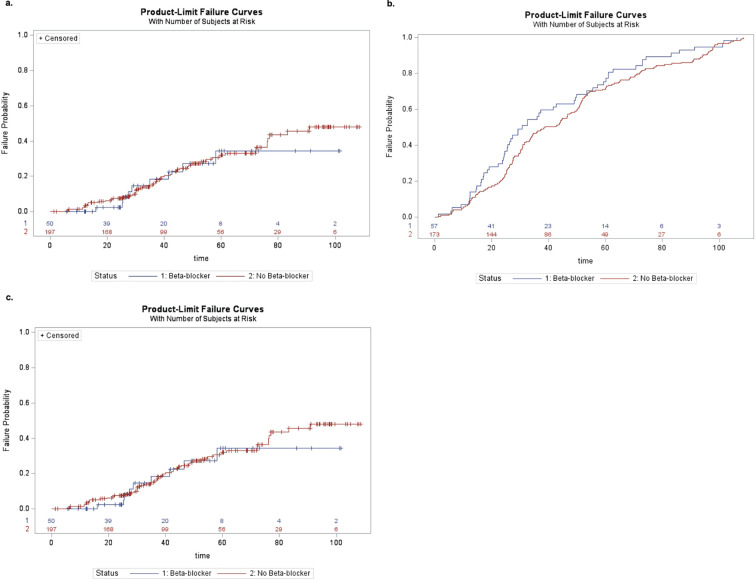
**(a)** Kaplan–Meier curve of conversion to any advanced AMD by beta-blocker use at enrollment. **(b)** Kaplan–Meier curve of conversion to neovascular AMD by beta-blocker use at the time of enrollment. **(c)** Kaplan–Meier curve of conversion to advanced non-neovascular AMD by beta-blocker use at the time of enrollment.

Kaplan-Meier curves are shown in **Figures 1a–c**. Univariate Cox proportional hazard modeling revealed a modestly significant difference in HR for conversion to NV AMD among beta-blocker users (HR: 1.92 [95% CI: 1.04, 3.55], *p* = 0.036) but no significant difference for conversion to any advanced AMD or advanced NNV (**Table 3**). However, in the adjusted model accounting for age and treated chronic hypertension, the risk of conversion to NV AMD was no longer significant. In the second set of models, where beta-blocker exposure was analyzed as a time-varying covariate to account for changes in beta-blocker status over the course of the study, no significant associations were found in either the univariate or multivariable models (**Table 4**). Survival analysis stratified by beta-blocker type also showed no significant differences in the risk of conversion to any advanced AMD (*p* = 0.8874).

**Table 3:** Cox proportional hazard model of beta-blocker status at enrollment and time to AMD conversion.

	Unadjusted model		Adjusted model*	
	HR (95% CI)	p-value	HR (95% CI)	p-value
Conversion to any advanced AMD	1.22 (0.78, 1.91)	0.382	1.14 (0.71, 1.84)	0.578
Conversion to NV	1.92 (1.04, 3.55)	0.036	1.62 (0.84, 3.10)	0.147
Conversion to advanced NNV	0.88 (0.44, 1.79)	0.732	0.86 (0.42, 1.79)	0.691

*Adjusted for age and chronic hypertension treatment.

**Table 4:** Cox proportional hazard model of time-varying beta-blocker use and time to AMD conversion.

	Unadjusted model		Adjusted model*	
	HR (95% CI)	p-value	HR (95% CI)	p-value
Conversion to any advanced AMD	0.96 (0.62, 1.50)	0.867	0.91 (0.57, 1.44)	0.682
Conversion to NV	1.61 (0.87, 2.97)	0.126	1.42 (0.75, 2.68)	0.286
Conversion to advanced NNV	0.70 (0.36, 1.36)	0.292	0.66 (0.34, 1.31)	0.238

*Adjusted for age and chronic hypertension treatment.

## Discussion

We examined the association between beta-blocker use and the risk of conversion to advanced AMD in an iAMD cohort followed longitudinally. In our univariate model, beta-blocker use was associated with a significantly increased hazard ratio for conversion to NV AMD. However, this association was no longer statistically significant in the adjusted model accounting for age and treated chronic hypertension. No significant associations were found between beta-blocker use and conversion to any advanced AMD or advanced NNV with beta-blocker use.

Vascular endothelial growth factor plays a central role in the pathogenesis of AMD, particularly advanced NV AMD. Beta-blockers have been associated with antiangiogenic effects, likely due in part to VEGF downregulation (19). In a mouse model, Lavine et al. investigated VEGF expression and laser-induced CNVM size following systemic administration of propranolol, a nonselective beta-blocker (7). They observed decreased VEGF expression and a 50% reduction in CNVM size in propranolol-treated mice. This provides compelling evidence for a VEGF-mediated pathway through which beta-blockade may be useful in treating NV AMD, though the findings are based on a small nonhuman study. However, several other studies challenge the idea that beta-blockers simply downregulate VEGF. For example, in the cardiac literature, beta-blockers have been shown to promote protective cardiac angiogenesis following ischemia, likely through increased VEGF—an effect that was negated when VEGF was blocked (20).

Given the evidence for beta-blockers’ role in VEGF regulation, they have potential for use in treating AMD. Adjunctive treatments combined with anti-VEGF therapy, for example, have been proposed as a way to enhance treatment efficacy and potentially reduce injection burden. Notably, a recent phase 1 clinical trial in Brazil examined the safety of intravitreal bevacizumab combined with propranolol, with published results confirming the safety of the drug combination (21). However, this possibility remains largely underexplored in both basic science and clinical research (22). In our study, we conducted a longitudinal analysis of patients with iAMD, comparing the risk of conversion to NV or advanced NNV AMD between those using and not using a beta-blocker. While beta-blockers are more directly implicated in VEGF-related NV AMD pathways, investigating their potential influence on NNV AMD may offer broader insights into AMD pathophysiology.

Although previous studies have examined this question, their findings have been largely inconsistent or contradictory (10–14), and most have been cross-sectional in nature, using variable methods to assess the association between beta-blockers and AMD. Traband et al. conducted a retrospective study comparing the mean number of intravitreal anti-VEGF injections in a cohort of NV patients on beta-blockers versus calcium channel blockers, while Kolomeyer et al. performed a similar study using the development of NV AMD as the endpoint—both found no significant effect of beta-blocker use (10, 15). Thomas et al. examined the use of several systemic medications—including beta-blockers, angiotensin-converting enzyme inhibitors, and angiotensin receptor blockers—among patients with NV AMD and those with NNV AMD, finding no difference in usage rates between the two groups (11). In a large cohort of hypertensive patients from the National Health and Nutrition Examination Survey, Luo et al. found that selective beta-blocker use was associated with higher odds of having any type of AMD in univariate analysis; however, this association was no longer significant after adjusting for confounding variables (13). They also found that nonselective beta-blockers were associated with lower odds of advanced AMD in multivariable analysis (13). Among patients with NV AMD, Montero et al. observed that those using a systemic beta-blocker required significantly fewer intravitreal injections on average compared to those not on a beta-blocker (*p* = 0.0068), albeit in a relatively small cohort of 46 patients (12).

One possible explanation for these inconsistent findings is that beta-blockers may influence AMD risk not only through a VEGF-mediated pathway but also via other causative mechanisms, such as blood pressure reduction. While we observed a significantly increased risk of conversion to advanced NV AMD among beta-blocker users, this association lost statistical significance after adjusting for age and chronic hypertension treatment history. The increased risk of conversion to NV AMD among beta-blocker users in our cohort was likely driven by hypertension-mediated effects, as adding HTN to the multivariable model altered the significance of beta-blockers. While the original Beaver Dam Eye Study did not identify hypertension as an AMD risk factor (3), numerous studies have suggested a potential link between hypertension and increased AMD risk (23–25). Compared to alternative medications, beta-blockers may help mitigate this association due to their antihypertensive and anti-VEGF properties, as shown in previous studies (26, 27). Further research is needed to determine how baseline hypertension influences the effectiveness of beta-blockers in slowing AMD progression. Some previous studies have adjusted for confounding factors, including hypertension, while others have not, which may partly explain past inconsistent findings.

Limitations of our study include the relatively small sample size, particularly the number of patients on specific beta-blocker medications, and the reliance on accurate charting of medication use at each clinic visit. Additionally, we did not capture the total duration of systemic beta-blocker exposure before study enrollment, limiting our ability to assess the effects of long-term use. The small sample size likely reduced our power to detect significant differences in conversion. Furthermore, similar to previous studies on AMD (10, 11), our study did not account for topical beta-blockers in categorizing patients’ beta-blocker use, as these medications have limited systemic absorption and minimal penetration into the posterior chamber of the eye (13, 14, 25, 26). Strengths of our study include the use of visit-by-visit medication data after enrollment and a meticulous review of multimodal images to retrospectively determine the time of conversion in the cohort. A large-scale prospective study that actively monitors their medication status may more effectively determine whether specific beta-blocker subtypes, such as nonselective versus selective, as well as variations in dose or frequency, influence AMD progression.

## Conclusion

In patients from the University of Colorado AMD Registry, systemic beta-blocker use was not significantly associated with the risk of conversion from intermediate to advanced NV and NNV AMD after adjusting for age and chronic hypertension treatment. In the unadjusted Cox proportional hazard model of beta-blocker status at enrollment and time to AMD conversion (**Table 3**), beta-blocker use showed a statistically significant reduction in conversion to NV AMD (*p* = 0.036), but this association became nonsignificant after adjustment.

## Data Availability

The datasets presented in this article are not readily available because they are currently not IRB approved for this purpose. Requests to access the datasets should be directed to Jennifer Patnaik at jennifer.patnaik@cuanschutz.edu.
